# Antitumor Activity of Nitazoxanide against Colon Cancers: Molecular Docking and Experimental Studies Based on Wnt/β-Catenin Signaling Inhibition

**DOI:** 10.3390/ijms22105213

**Published:** 2021-05-14

**Authors:** Noha M. Abd El-Fadeal, Mohamed S. Nafie, Mohammed K. El-kherbetawy, Amr El-mistekawy, Hala M. F. Mohammad, Alaaeldeen M. Elbahaie, Abdullah A. Hashish, Suliman Y. Alomar, Sheka Yagub Aloyouni, Mohamed El-dosoky, Khaled M. Morsy, Sawsan A. Zaitone

**Affiliations:** 1 Department of Medical Biochemistry and Molecular Biology, Faculty of Medicine, Suez Canal University, Ismailia 41522, Egypt; Noha_abdelfadeal@med.suez.edu.eg; 2 Chemistry Department, Faculty of Science, Suez Canal University, Ismailia 41522, Egypt; mohamed_nafie@science.suez.edu.eg; 3 Department of Pathology, Faculty of Medicine, Suez Canal University, Ismailia 41522, Egypt; Mohamed_elkherbetawy@med.suez.edu.eg; 4 Department of Internal Medicine, Gastroenterology Division, Faculty of Medicine, Al-azhar University, Cairo 11651, Egypt; drmistekawy@azhar.edu.eg; 5 Department of Clinical Pharmacology, Faculty of Medicine, Suez Canal University, Ismailia 41522, Egypt; hala_mohamed@med.suez.edu.eg; 6 Central Laboratory, Center of Excellence in Molecular and Cellular Medicine (CEMCM), Faculty of Medicine, Suez Canal University, Ismailia 41522, Egypt; 7 Department of Clinical Oncology and Nuclear Medicine, Faculty of Medicine, Suez Canal University, Ismailia 41522, Egypt; alaaeldeen_mahmoud1@med.suez.edu.eg; 8 Basic Medical Sciences Department, College of Medicine, University of Bisha, Bisha 61922, Saudi Arabia; aahashish80@med.suez.edu.eg; 9 Department of Clinical Pathology, Faculty of Medicine, Suez Canal University, Ismailia 41522, Egypt; 10 Doping Research Chair, Department of Zoology, College of Science, King Saud University, Riyadh 11495, Saudi Arabia; 11 Health Sciences Research Center, Princess Nourah bint Abdulrahman University, Riyadh 36285, Saudi Arabia; syaloyouni@pnu.edu.sa; 12 Department of Neuroscience Technology, College of Applied Medical Sciences in Jubail, Imam Abdulrahman Bin Faisal University, Jubail 35816, Saudi Arabia; mesalama@iau.edu.sa; 13 Department of Anesthesia Technology, College of Applied Medical Science in Jubail, Imam Abdulrahman Bin Faisal University, Jubail 35816, Saudi Arabia; kmibraheem@iau.edu.sa; 14 Department of Pharmacology and Toxicology, Faculty of Pharmacy, Suez Canal University, Ismailia 41522, Egypt; 15 Department of Pharmacology and Toxicology, Faculty of Pharmacy, University of Tabuk, Tabuk 714, Saudi Arabia

**Keywords:** apoptosis, mouse colon cancer, molecular docking, nitazoxanide, PCNA, Wnt/β-catenin signaling

## Abstract

In colon cancer, wingless (Wnt)/β-catenin signaling is frequently upregulated; however, the creation of a molecular therapeutic agent targeting this pathway is still under investigation. This research aimed to study how nitazoxanide can affect Wnt/β-catenin signaling in colon cancer cells (HCT-116) and a mouse colon cancer model. Our study included 2 experiments; the first was to test the cytotoxic activity of nitazoxanide in an in vitro study on a colon cancer cell line (HCT-116) versus normal colon cells (FHC) and to highlight the proapoptotic effect by MTT assay, flow cytometry and real-time polymerase chain reaction (RT-PCR). The second experiment tested the in vivo cytotoxic effect of nitazoxanide against 1,2-dimethylhydrazine (DMH) prompted cancer in mice. Mice were grouped as saline, DMH control and DMH + nitazoxanide [100 or 200 mg per kg]. Colon levels of Wnt and β-catenin proteins were assessed by Western blotting while proliferation was measured via immunostaining for proliferating cell nuclear antigen (PCNA). Treating HCT-116 cells with nitazoxanide (inhibitory concentration 50 (IC50) = 11.07 µM) revealed that it has a more cytotoxic effect when compared to 5-flurouracil (IC50 = 11.36 µM). Moreover, it showed relatively high IC50 value (non-cytotoxic) against the normal colon cells. Nitazoxanide induced apoptosis by 15.86-fold compared to control and arrested the cell cycle. Furthermore, nitazoxanide upregulated proapoptotic proteins (P53 and BAX) and caspases but downregulated BCL-2. Nitazoxanide downregulated Wnt/β-catenin/glycogen synthase kinase-3β (GSK-3β) signaling and PCNA staining in the current mouse model. Hence, our findings highlighted the cytotoxic effect of nitazoxanide and pointed out the effect on Wnt/β-catenin/GSK-3β signaling.

## 1. Introduction

According to the Global Cancer Repository 2018 records, colon cancer is the third most deadly cancer in the world [1]. Recent years have seen very significant progress in the field of treatment of this debilitating disease. Many studies revealed the role of various mediators in almost all steps of colon tumorigenesis, including initiation, promotion, progression, and metastasis [2].

The development of gene mutations is thought to be the primary motivating factor in the early stages of colon cancer [3] such as genetic changes related to the wingless (Wnt)/β-catenin signaling. The latter pathway is a distinctive signaling pathway that controls gene expression, migration, proliferation, and differentiation of colon cancer [3]. β-catenin, the key intermediate for Wnt signaling, is found in adherent junctions, in the cytoplasm and also in the nucleus; in these cellular compartments, it controls several biological interactions [4]. A high level of nuclear β-catenin is linked to poor prognosis in colon cancer patients [5] and increased susceptibility to cancer recurrence and decreased survival rate [6]. Among the molecular regulators of the Wnt signaling is the glycogen synthase kinase-3β (GSK-3β) [7] which is considered as a multifunctional serine/threonine kinase that controls a variety of cellular pathways [8,9]. Importantly, overexpression of Wnt or β-catenin proteins was reported to induce the expression of proliferating cell nuclear antigen (PCNA) [10]. 

Nitazoxanide [2-(acetyloxy)-N-(5-nitro-2-thiazolyl)benzamide] is an antiparasite medication marketed by Romark Laboratories in 2002 [11]. Nitazoxanide’s anti-cancer reactivity was reported in some articles [12]. Nitazoxanide provided high anti-cancer activity in various cancer cells [13] and tumor models [12,14]. The anti-cancer effect of nitazoxanide is explained by different actions including autophagy and anti-cytokine activity [15]. Pharmacokinetic properties of nitazoxanide involve oral absorption and hydrolysis to its active metabolite, tizoxanide, which conjugates with glucuronide. Nitazoxanide is largely well tolerated with no serious adverse effects for human [16].

It is important to find molecules and signaling pathways that are crucial for colon cancer and to create novel medications to target them. Wnt signaling was recently considered as a target for treating colon cancer. The current study was planned to test the cytotoxic activity of nitazoxanide in an in vitro colon cancer cell line and in vivo chemically induced colon cancer. Furthermore, the scope of this paper was extended for exploring the impact of nitazoxanide on Wnt/β-catenin signaling and tumor apoptosis.

## 2. Results

### 2.1. Experiment 1: In Vitro Cytotoxic Activity

#### 2.1.1. Cytotoxic Activity against Colon Cancer Cells and Normal Colon Cells

**Figure 1:**
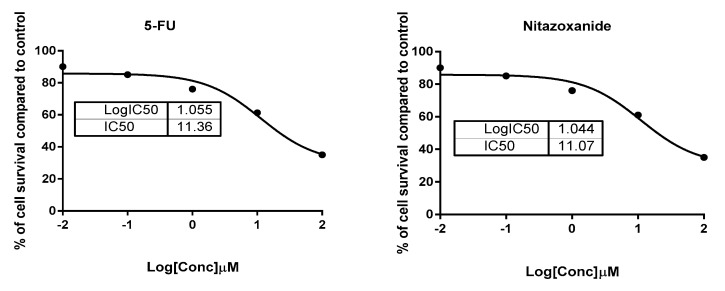
Percentages of cell viability of HCT116 cells treated with nitazoxanide using serial dilutions from 100 to 0.01 µM.

Testing nitazoxanide for its cytotoxic activity against HCT-116 colon cancer cells and FHC (normal colon cells) is shown in Table 1. The MTT results demonstrated potent cytotoxic activity of nitazoxanide against the HCT-116 with the IC50 value of 11.07 µM against the value recorded with 5-flurouracil (5-FU, IC50 = 11.36 µM), so the compound was more cytotoxic than the 5-FU (Figure 1). Additionally, it was safe against the FHC with a relatively high IC50 value of 48.4 µM. Hence, it was concluded the activity and the selectivity of nitazoxanide in its action. Hence, it was tested for its apoptotic activity using flow cytometric analysis and the gene expression level.

#### 2.1.2. Exploration of Apoptosis Indicators

##### Annexin/Propidium Iodide (PI) Staining and Cell-Cycle Analysis

**Figure 2:**
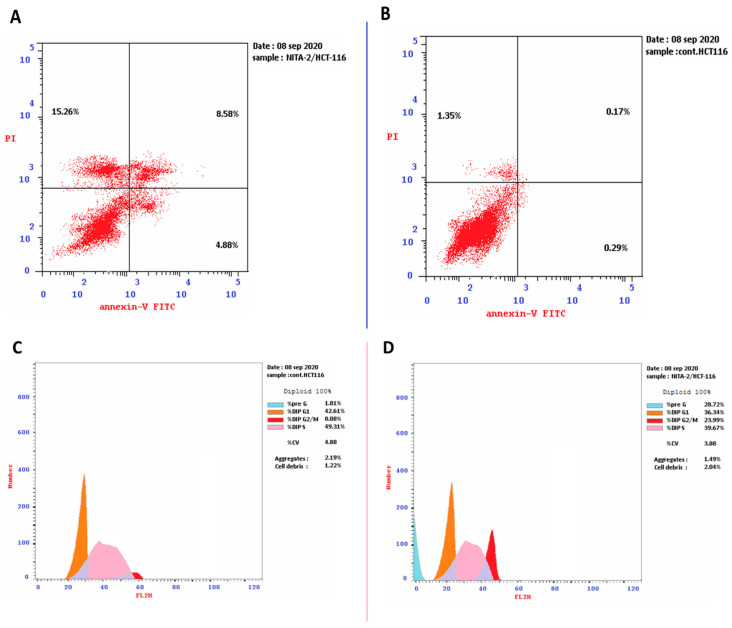
Cryptographs of annexin-V/propidium iodide staining. (**A**): untreated and (**B**): treated HCT-116 cells showing the apoptotic effect of nitazoxanide (IC50 = 11.07 μM, 48 h) in treated cells. Also, histograms DNA content-flow cytometry aided cell cycle analysis of (**C**): untreated and (**D**): treated HCT-116 cells with compound Nitazoxanide (IC50 = 11.07 μM) for 48 h of incubation.

Nitazoxanide (IC50 = 11.07 μM) was added to the HCT-116 cancer cells for 48 h. We investigated its apoptotic activity using the cell cycle analysis. Nitazoxanide significantly increased apoptotic cell death with 15.86-fold (28.72% versus 1.81% for the control). It encouraged the early, intermediate, and late apoptotic cell death by 4.88%, 15.26%, and 8.58%, respectively. Moreover, DNA flow cytometry was employed for analysing the kinetics of the cell cycle in HCT-116 cancer cells treated with nitazoxanide. As seen in Figure 2, nitazoxanide significantly increased cell population at the G2/M cell phase (23.99% versus 8.08% in the control test) and the cell population in the %Pre-G1 (28.72% compared to 1.81% in the control test). However, nitazoxanide non-significantly decreased the cell population in both phases S% (39.67% reduction compared to 49.31% for control) and G0/G1 by (36.34% reduction compared to 42.61% for control). Thus, we can conclude that nitazoxanide prompted the arrest of the pre-G1 and G2/M-phase cell cycle and inhibited the HCT-116 cancer cells progression. 

##### String Database Query Methods and Real-Time Polymerase Chain Reaction (RT-PCR)

**Figure 3:**
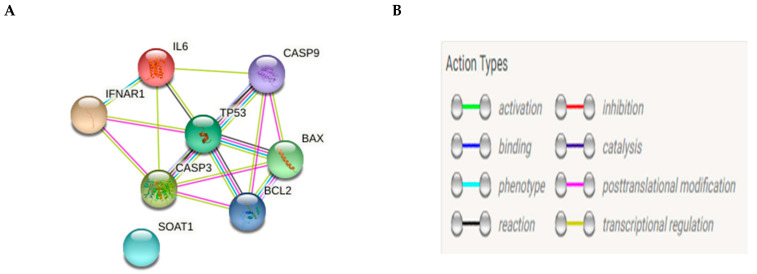
Network analysis of pro-apoptotic and anti-apototic genes (**A**) and the action types (**B**).

After accessing the website (http://string-db.org, accessed on 10 April 2021), we selected multiple protein query and wrote our desired set of proteins (p53, BAX, BCL2, caspase-3, caspase-8, caspase-9) sequentially then; we got a network view (Figure 3A) using the default setting to inspect cross-links weighted scheme to rank matched annotated proteins and finally, we collected evidence about our protein’s interaction by the linked colored lines between the proteins, as shown in (Figure 3B).

**Figure 4:**
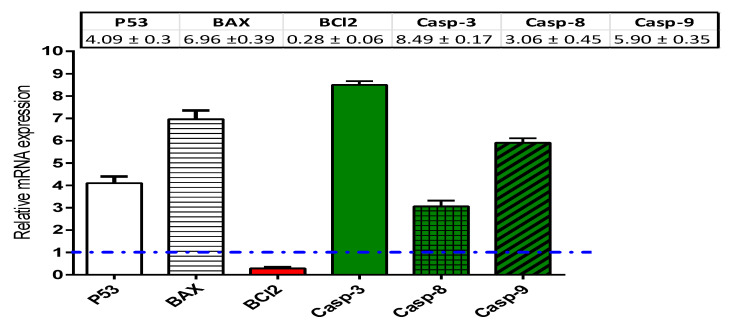
Real-time polymerase chain reaction (RT-PCR) analysis of the apoptosis genes. Assay was done using HCT-116 cells incubated with the IC50 of nitazoxanide. Dashed line represents the control (Fold change = 1), while β-actin as a housekeeping gene. ΔCT; the difference between the CT values of target genes and the housekeeping one (β-actin). ΔΔCT; The difference between mean values of genes CT values in the treated and control groups. Fold of change is an exponential function represented by 2^–ΔΔCT.

HCT-116 cells treated with nitazoxanide (IC50 = 11.07 μM) for 48 h were used for performing the real-time polymerase chain reaction (RT-PCR) assays. In HCT-116 cells, the mRNA expression of the pro-apoptotic proteins (P53 and BAX), caspases (-3, -8, and -9), and the anti-apoptotic protein (BCL-2) was measured. Nitazoxanide significantly activated the mRNA expression of P53 (≈4.09-fold) and BCL-2-associated X protein (BAX, 6.96-fold). Further, nitazoxanide significantly increased the mRNA levels of caspases 3 (≈8.49-fold), 8 (3.06-fold), and 9 (5.90-fold) genes. By contrast, nitazoxanide significantly inhibited the BCL-2 mRNA expression (≈0.28-fold) (Figure 4).

### 2.2. Experiment 2: In Vivo Antitumor Activity

#### 2.2.1. Pathway Enrichment Analysis

**Figure 5:**
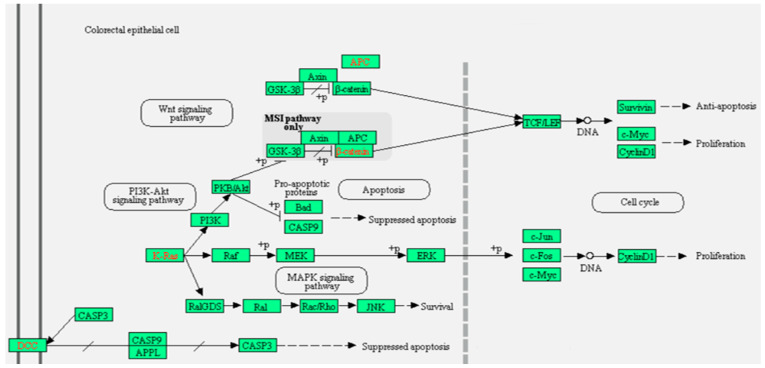
Colorectal cancer Wnt signaling pathway. Graph created by KEGG and Reactome bioinformatic databases.

Using the online bioinformatic KEGG and Reactome databases we found that Wnt signaling regulates various developmental and adult process containing stem cell maintenance, cell proliferation, and cell death [17,18]. Wnt protein activates a transcriptional cascade through binding to 1–10 humans Frizzled (FZD) receptors in combination with the LRP5/6 co-receptors; hence it regulates many functions like proliferation and stem cell self-renewal. Engagement of the FZD-LRP receptor by Wnt ligand leads to stabilizing and translocating the cytosolic β-catenin to enter the nuclei. In the nucleus, β-catenin performs as a co-activating factor for transcription dependent on lymphoid enhancer-binding factor and T cell factor. However, when Wnt ligand is absent, the cytosolic β-catenin is phosphorylated by a degradation complex containing GSK-3β, casein kinase 1, axin, and adenomatous polyposis coli (APC) and afterward subjected to ubiquitination and degradation by the 26S proteasome [17,19] (Figure 5).

In the planar cell polarity (PCP) pathway, Wnt ligand binds to the FZD receptor leading to stimulation of small Rho GTPases and JNK. The later enzymes control the cytoskeleton and manage cell migration and polarity [20,21]. In some circumstances, a FZD-Wnt interaction upsurges intracellular calcium level and stimulates Ca2+/calmodulin-dependent protein kinase (CaMK) II and *protein kinase C*; this Wnt calcium pathway stimulates cell migration and prevents the canonical β-catenin reliant transcriptional pathway [22,23,24]. When Wnt binds to tyrosine kinase-like orphan *receptors* or RYK receptors, it controls cell migration (Figure 5).

#### 2.2.2. Effect of Nitazoxanide on the Levels of Wnt/Catenin/Glycogen Synthase Kinase-3β (GSK-3β) Proteins in Colon Cancer Model

**Figure 6:**
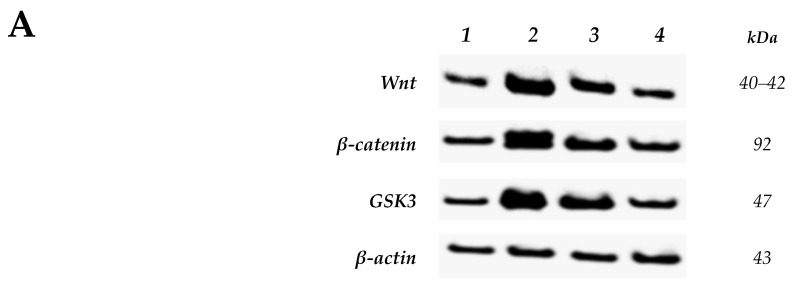

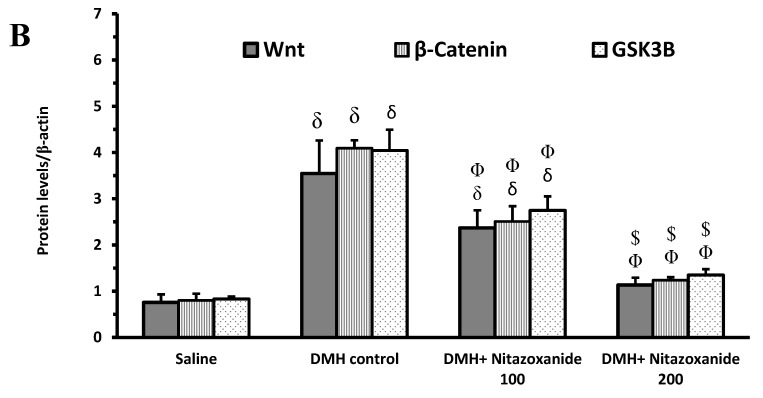
Western blot analysis for Wnt/β-catenin/glycogen synthase kinase-3β (GSK3) proteins. (**A**): Western blot gels of the target genes in (1) saline group, (2) 1,2-dimethylhydrazine (DMH) control group, (3&4) DMH + nitazoxanide 100 and 200 mg/kg groups, (**B**): Column charts for Wnt, β-catenin and GSK3B proteins relative to β-actin. Data are mean ± standard deviation (SD) and analyzed by one-way analysis of variance (ANOVA) and Bonferroni’s test. ^δ^ Compared to saline group, ^Φ^ Compared to DMH control group, $Compared to DMH + nitazoxanide 100 group, *p* < 0.05.

Figure 6A demonstrates the WB protein levels normalized to β-actin. Images show decreased Wnt, β-catenin and GSK-3β proteins in the nitazoxanide treated groups (100 and 200 mg) and this was demonstrated by the thinner bands in a Western blot (Figure 6A). The measured density for Wnt, β-catenin, and GSK-3β protein bands were found decreased in nitazoxanide treated group than the 1,2-dimethyhydrazine (DMH) control group (Figure 6B–D). 

#### 2.2.3. Effect of Nitazoxanide on Pathologic Picture in Mice with Colon Cancer 

**Figure 7:**
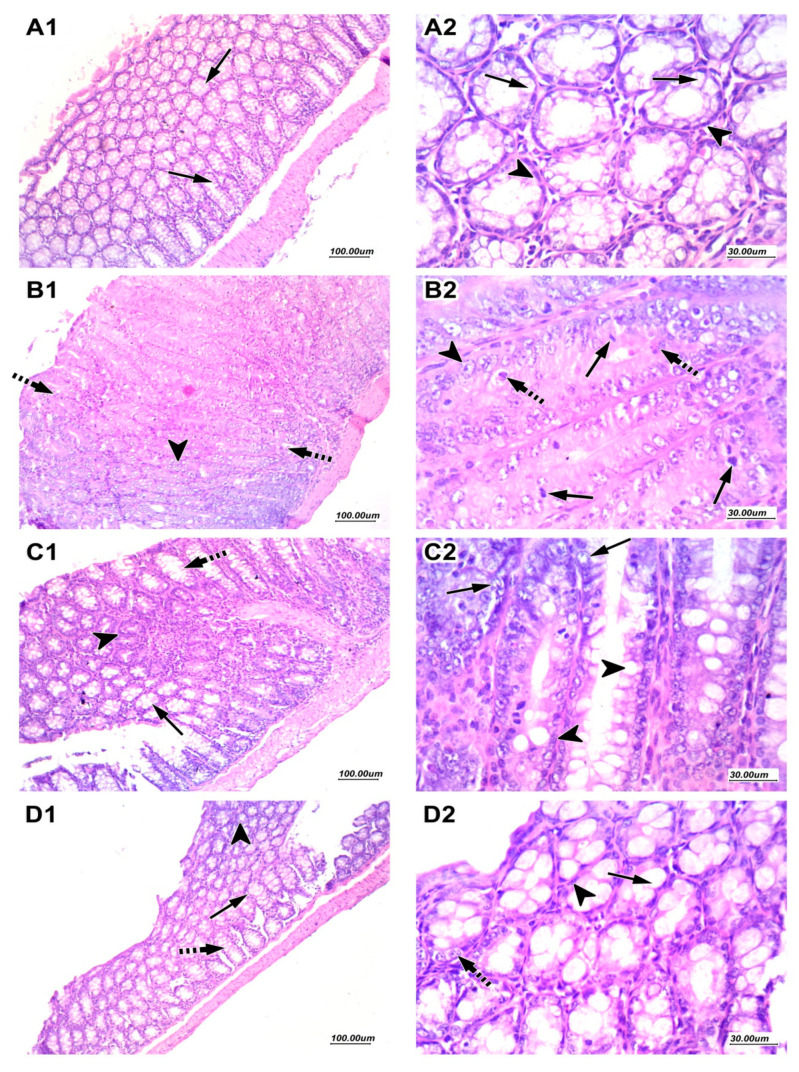
Histopathological picture for colon samples stained with hematoxylin and eosin (H&E). (**A1**) and (**A2**) images of saline group showed regular mucus secreting glands with preserved goblet cells (arrow) and basal flattened nuclei (arrowhead). (**B1**) and (**B2**) images of DMH control group showed nuclear enlargement with hyperchromasia (arrowhead) with loss of mucus secretion and prominent mitotic figures (arrow) with apoptotic bodies (dashed arrow). (**C1**) and (**C2**) images of nitazoxanide 100 treated group showed epithelial cells with mucus-secreting activity (arrow) and few scattered cells showing dysplastic changes (arrowhead). (**D1**) and (**D2**) images of nitazoxanide 200 treated group revealed marked improvement with regular mucus-secreting epithelial cells (arrow) with very few cells showing slight nuclear enlargement (dashed arrow). H&E, (**A1**, **B1**, **C1** & **D1**: 100×, **A2**, **B2**, **C2** & **D2**: 400×).

Hematoxylin and eosin (H&E)-stained sections of the saline group at low magnification (100×) showed regularly arranged colonic crypts with preserved goblet cells. High magnification (400×) showed a regular arrangement of epithelial cells with oval to flattened basal nuclei with regular nuclear membrane, fine chromatin, and cytoplasmic luminal mucin secretions (Figure 7A1 and Figure 6A2).

Sections from mice in the DMH control group showed markedly crowded distorted colonic crypts with an almost total loss of goblet cells with prominent cellular crowding, enlarged hyperchromatic nuclei with many scattered mitotic figures and apoptotic bodies, changes appreciated in the full thickness of mucosa from the surface to base (Figure 6B1,B2). Sections from nitazoxanide 100 treated group showed decreased crypt distortion with restored goblet cells and mucus secreting activity in many crypts. Few scattered crypts showed focal dysplastic changes (Figure 7C1 and Figure 6C2). Sections from Nitazoxanide 200 treated group showed predominantly regularly arranged crypts with presented goblet cells and mucus secreting activity. Very Few cells showed slightly enlarged nuclei (Figure 7D1,D2).

**Figure 8:**
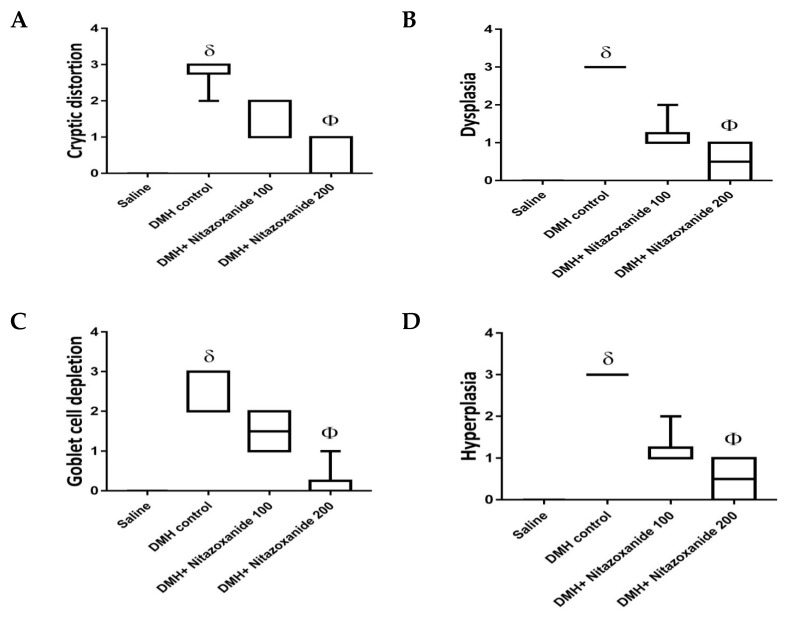

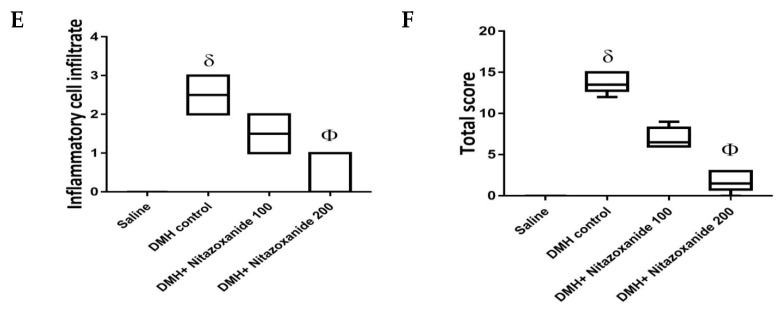
Histopathologic score for colon specimens stained with routine hematoxylin and eosin staining. Scores from 0–3 were assigned to each microscopic field for (**A**) Cryptic distortion, (**B**) Dysplasia, (**C**) Goblet cell depletion, (**D**) Hyperplasia, (**E**) Inflammatory cell infiltrates and (**F**) total histologic score. Data are box-and-whisker plots for the scoring values for 5 fields/colon section. ^δ^ Compared to saline group, ^Φ^ Compared to DMH control group, *p* < 0.05.

Histopathologic scoring data are demonstrated in Figure 8 and classified as cryptic distortion, dysplasia, goblet cell depletion, hyperplasia, inflammatory cell infiltrates as well as the sum of the 5 scores. The DMH control group showed significant increases in all the created individual scores and the total score compared to the saline group. Importantly, DMH+ nitazoxanide 200 mg/kg group showed significant reductions in all the 5 individual scores as well as the total histologic score (Figure 8A–F).

#### 2.2.4. Effect of Nitazoxanide on Proliferating Cell Nuclear Antigen (PCNA) Immunoractivity in Mice with Colon Cancer

**Figure 9:**
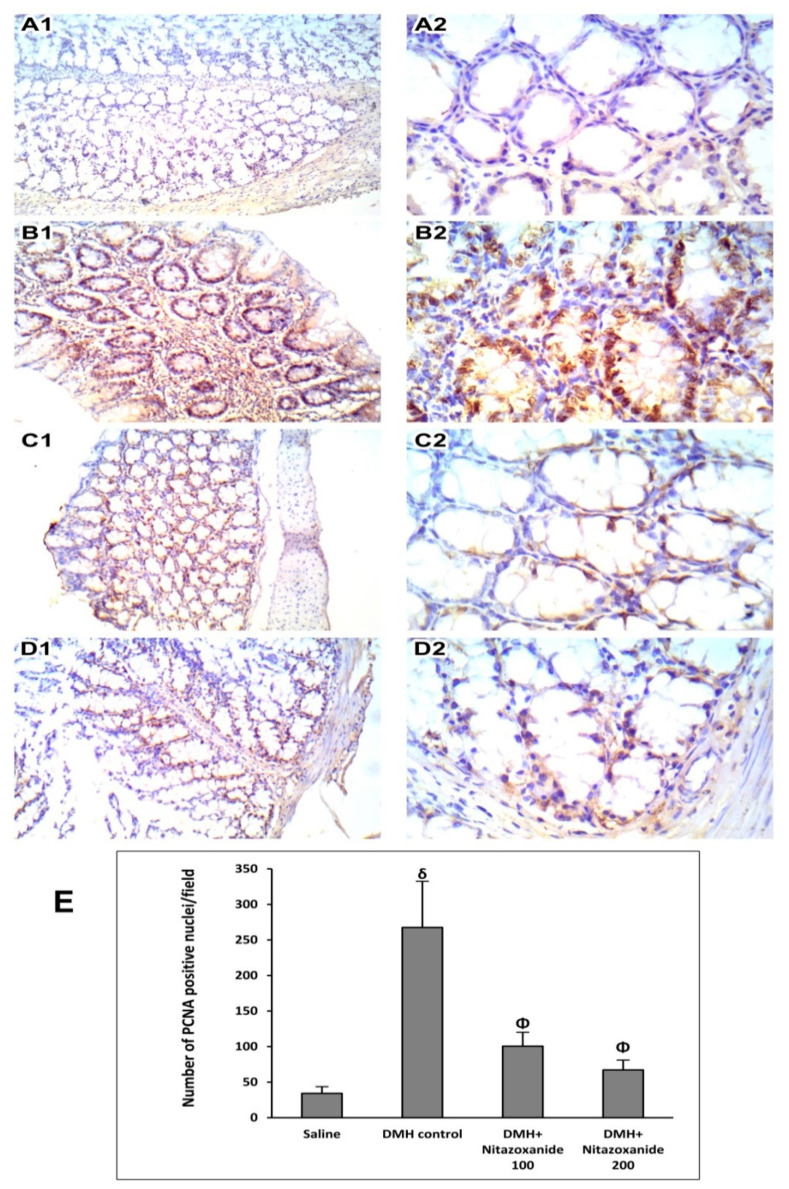
Immunohistochemistry for proliferating cell nuclear antigen (PCNA) in colon samples. (**A1**) and (**A2**) images of saline group showed regular mucus and mild staining and basal flattened nuclei. (**B1**) and (**B2**) images of DMH control group showed high nuclear staining. (**C1**) and (**C2**) images of nitazoxanide 100 mg/kg treated group showing moderate PCNA staining. (**D1**) and (**D2**) images of nitazoxanide 200 treated group low staining for PCNA. PCNA immunohistochemistry, (**A1**, **B1**, **C1** and **D1**: 100×, **A2**, **B2**, **C2** and **D2**: 400×). (E) Column chart for the number of PCNA positive nuclei per field, 5 random fields per tissue section. ^δ^ Compared to saline group, ^Φ^ Compared to DMH control group, *p* < 0.05.

PCNA immunostained sections of the saline group showed scattered positive cells in the crypt base indicating low proliferative activity (Figure 9A1,A2). Sections from mice in DMH control group showed markedly increased proliferative activity with numerous positive nuclei at the crypt base and superficial part (Figure 9B1,B2). Sections from the nitazoxanide 100 treated group showed decreased proliferative activity with few cells showing nuclear positivity at crypt bases (Figure 9C1,C2). Sections from the nitazoxanide 200-treated group showed scattered few cells showing nuclear positivity at the crypt base (Figure 9D1,D2). Panel 8E demonstrates the mean of the number of PCNA positive nuclei in colon specimens of the study groups. The DMH control group showed significant elevation in the number of PCNA positive nuclei while mice groups treated with nitazoxanide 100 mg/kg or nitazoxanide 200 mg/kg showed significant reductions in the PCNA staining (Figure 9E).

### 2.3. Experiment 3: In Silico Mechanism of Action Simulations

We tried to elucidate the simulated mechanism of nitazoxanide binding and used the structure-based drug design tool to dock it inside the β-catenin protein with protein data bank code (PDB = 3SL9) to validate the β-catenin signaling pathway. Full analysis of drug–target interactions of nitazoxanide, with binding energies, 2D and 3D interactions are summarized in Table 2. Nitazoxanide was docked within the β-catenin protein and the binding energy was −10.58 Kcal/mol, and it formed two hydrogen bonds (HB) interactions through the two carbonyl groups as hydrogen bonding acceptor with Asn 290 as the key interacting amino acid (Table 2).

## 3. Discussion

It is known that nitazoxanide was created first as an antiprotozoal drug and approved for treating the *Cryptosporidium parvum* and *Giardia lamblia* that infest the intestine [25]. Studies also demonstrated that nitazoxanide displays a broad spectrum activities against viruses, bacteria, parasites and cancers [16,26], however the pathway that being targeted by nitazoxanide in human cells was previously known a little. 

In the current study, nitazoxanide was tested for its cytotoxic activity in 2 models of colon cancer (in vitro and in vivo models) and some mechanistic approaches were tested. The influence of nitazoxanide on some apoptosis genes including BAX, P53, caspase (-3, -8 and -9), and BCL-2 was tested in vitro while its effect on Wnt/β-catenin/GSK-3β proteins was tested in vivo. The latter was confirmed with a molecular docking study.

We believe that String database extract curated data using the following sources: (1) systematic co-expression analysis, (2) detection of shared selective signals across genomes, (3) automated text-mining of the scientific literature and (4) computational transfer of interaction knowledge between organisms based on gene ortholog. Hence, STRING analysis was conducted to confirm that the selection of our gene’s expression in HCT116 work beside some other genes that may participate in the whole process.

Interestingly, in the current in vitro study, a 48-h incubation period with nitazoxanide’s IC50 increased the percentage of apoptotic cells in colon cancer cell line. Further, colon cancer cells showed greater mRNA expression for the pro-apoptosis proteins like P53, BAX, and caspases (-3, -8 and, -9) but lower mRNA expression for BCL-2. These genes interplay and the interaction was shown using the STRING bioinformatics database. These findings were in accordance with the network analysis using STRING. Apoptosis is known as the programmed cell death. During apoptosis, numerous extrinsic factors influence p53. The active p53 undergoes attachment to an exact sequence in the DNA causing transcriptional activation of many genes involved in apoptosis as BCL-2 proteins family. In addition, BAX changes the anti-apoptotic effect of BCL-2 protein resulting in release of cystolic mitochondrial cytochrome c of colon cancer human cells. The release of cytochrome c is further managed via additional intrinsic activators of the BCL-2 proteins family [27].

In accordance with the present results, nitazoxanide was documented to diminish the growth of the tumor by c-Myc inhibition and inducing apoptosis in breast cancer xenografts in mice [15]. Furthermore, the anti-cancer properties of nitazoxanide have been examined and validated previously in human colon cancer cell lines [28] and animal models [15]. Nitazoxanide hinders critical metabolic and pro-death signals like autophagy, cause unfolding for protein response, autophagy, anti-cytokine actions and inhibition of c-Myc [16]. A non-oncologic experiment showed that nitazoxanide possesses a direct inhibitory effect on IL-6 production in vitro and in vivo mice models [29]. However, the mechanism by which nitazoxanide was able to block IL-6 production is not known [16]. 

In the current study, Wnt/β-catenin/GSK-3β proteins were upregulated in the in vivo chemically induced colon cancer in mice. Wnt signaling is considered as one of the highly conserved pathways that perform many important regulatory roles such as tissue homeostasis and biological processes The Wnt signaling pathways are subdivided into the non-canonical β-catenin-independent and the canonical β-catenin-dependent pathways. The latter performs balancing roles in physiologic functions and pathologic features of the adult intestine for example, it preserves the crypt stem cell compartments in health. When it is stimulated by mutation, it enhances the development of colon cancer [30]. The current results agree with those documented by Senkowski et al. [31]. In agreement, aberrant Wnt/β-catenin signaling in colon cancer promotes chromosomal instability [32,33]. Evidence came from observing downregulation in DICKKOPF-1 gene which is a Wnt antagonist in colon cancer [34]. 

In the in vivo colon cancer experiment, nitazoxanide triggered a dose-dependent repression in Wnt/β-catenin/GSK-3β protein production. These reductions were followed by a change in the production of PCNA antigen; a downstream target of β-catenin. Overall, these data are supported by previous findings that demonstrated an inhibitory effect for nitazoxanide on colon cancer progression by abrogating β-catenin [35,36]. 

In the current study, nitazoxanide downregulated the colon production of Wnt and β-catenin protein. One paper reported that nitazoxanide activates the 5’ AMP-activated protein kinase (AMPK) signaling and downregulate the mechanistic target of rapamycin (mTOR) and Wnt signaling in a cell culture based screening; authors claimed that this action occurred at clinically achievable concentrations [37]. The probable mechanism may be due to the increased citrullination caused by nitazoxanide and there resulting stabilizing peptidyl arginine deiminase type-2 that finally causes degradation of β-catenin. 

Moreover, our results revealed that GSK-3β is downregulated by nitazoxanide as demonstrated by Western blotting assay. It was previously documented that GSK-3β has different effects on cancer cells [38,39]. GSK-3β negotiates the nuclear factor-ҡ (NF-ҡB) pathway and so it plays a vital role in cell survival [40,41] specifically in colon cancer in which there is coactivation for both Wnt/β-catenin and NF-ҡB pathways through ubiquitin system dysregulation [42]. Some studies revealed that treating colon cancer cell lines with various concentrations of the GSK-3β inhibitors reduced the viability of cells and stimulated apoptotic machinery in a dose-dependent way [43,44,45]. 

## 4. Materials and Methods

### 4.1. Medications

5-Flurouracil (5-FU) vials were purchased from the Merck Company (Cat. No. 343922, Merck Company, Branchburg, NJ, USA) whereas nitazoxanide was provided by Unidrug Innovation Pharma Technologies Ltd. (Batch No. NTZ/1603014, Analytical reference #QNTZ/16014, Indore, India) and dissolved in distilled water. 

### 4.2. Experiment 1: In Vitro Experiment

#### 4.2.1. Cell Culturing and Cytotoxic Activity Using the MTT Assay

Both normal colon (FHC; ATCC^®^ CRL-1831™) and colon cancer (HCT-116; ATCC^®^ CCL-247™)) cells were procured from the American Type Culture Collection (ATTC) and maintained in Dulbecco’s Modified Eagle Medium/F-12 (DMEM⁄F12; cat no. D0547.DMEM⁄F12, Sigma-Aldrich, Saint Louis, MO, USA). Both cell types were provided with 2 mM L-glutamine (Catalog #: BE17-605E Lonza, Bornem, Belgium) and 10% fetal bovine serum (FBS) purchased from (CAT.NO.12103C Sigma-Aldrich, St. Louis, MO, USA), 1% penicillin/streptomycin (Lonza, Belgium). Cell incubation was done at 37 °C in a 5% carbon dioxide atmosphere (NuAire). Cell plating at a density equal to 5000 cells was undertaken in triplicate in a plate of 96 wells [46]. On the second day, cells were treated with 5-FU or nitazoxanide at the (0.01, 0.1, 1, 10, and 100 µM) concentrations. Cell viability was assessed after 48 h using MTT solution (Promega, Madison, WI, USA) [47]. 20 μL of MTT dye were transferred to wells and then incubation of the plate was allowed for a period of three hours. Colour intensity was subsequently read at 570 nm employing a BIO-Rad enzyme-linked immunosorbent assay (ELISA) microplate reader (iMark™ #1681130, BIO-Rad, CA, USA). The viability was calculated relative to a control and the IC50 values were determined using the GraphPad prism 7 as previously reported [48,49].

#### 4.2.2. Investigation of Apoptosis in the Colon Cancer Cells

This routine work for apoptosis investigation was undertaken only in the HCT-116 cells, not the normal cells. As the IC50 for nitazoxanide was >48 µm, so nitazoxanide was not considered cytotoxic to normal colon cells, therefore, we thought there was no need to investigate apoptosis markers.

##### Annexin V/PI Staining and Cell-Cycle Analysi

Analysis of the cell cycle is a key test for determining each phase cell population when treated with cytotoxic substances. Apoptosis rate in the colon cancer cells (HCT116) was quantified using annexin V-FITC (V-fluorescein isothiocyanate) (BD Pharmingen, San Diego, CA, USA). Cells were transferred to 6-well culturing plates (3–5 × 10^5^ cells per well) and incubated overnight. Cells were then treated with nitazoxanide for 48 h. Next, media supernatants and cells were rinsed with ice-cold phosphate-buffered saline (PBS). The next step was suspending the cells in 100 µL of annexin binding buffer containing 1.4 M NaCl, 25 mM CaCl_2_ and 0.1 M HEPES/NaOH (final pH equals 7.4) and then incubated with 1:100 5-FU annexin V-FITC solution and 10 µg/mL propidium iodide (PI; Sigma, St. Louis, MO, USA) in the dark for half an hour. Stained cells were then measured by a Cytoflex flow cytometer (Beckman Coulter Inc., California, USA). Data were analyzed using cytExpert software (V2.4) [50,51].

#### 4.2.3. Real Time-Polymerase Chain Reaction for the Selected Genes

HCT-116 cells were treated with nitazoxanide (IC50 = 11.07 μM) for 48 h. After completing the treatment period, total cell RNA was extracted utilizing Qiagen Rneasy^®^ Mini Kit (cat.no. 74104, Hilden, Germany). Then, 500 ng of RNA were used to synthetize cDNA by utilizing *i*-Script cDNA synthesis kit from BioRad (cat.no. 1708891, Hercules, USA). Finally, each RT-PCR reaction was performed using 25 µL of Fluocycle^®^II SYBR^®^ purchased from Euroclone (cat.no.ERD002100BIM, Milan, Italy), 10 ng of cDNA and 2 μL from forward and reverse primers (prepared at 10 µM solutions). We completed the reaction mix by adding 19 μL of nuclease free water. RT- PCR using StepOne™ Real-Time PCR (cat.no.: 4376357, Foster City, USA). The real-time PCR instrument was adjusted on the following cycling conditions: denaturation at 95 °C for a 5-min period; 35 cycles of 95 °C for 15 s, 51–60 °C cycles for 30 s according to the assessed target gene, and 72 °C for 60 s [50,52]. Then, the Ct values were collected for the calculation of the relative genes’ expression in all samples by normalization to the β-actin housekeeping gene [53]. According to previous studies, the expression of β-actin is assumed to remain constant and useful for normalization for variations in signal quantification [54,55]. The specificity of the PCR analysis was determined by the melting curve and gel electrophoresis. Table 3 demonstrates the sequences of the gene primers. 

### 4.3. Experiment 2: In Vivo Experiment

#### 4.3.1. Ethics Statement

The experimental protocol was permitted by the Research Ethics Committee (approval number 201907RA5, Faculty of Pharmacy, Suez Canal University).

#### 4.3.2. Animal Environment

Hamada Abdelhaleem Company in Giza (Egypt) supplied our animal house with male Swiss albino mice (Body weigh range was 21–28 g). The mice were maintained under a normal day/night cycle and hygienic environment. Mice were allowed to acclimatize to the study conditions for 12 days prior to experimentation. Basal diet and water were provided ad libitum.

#### 4.3.3. Induction of Colonic Cancer and Experimental Groups

DMH was procured from Sigma-Aldrich (USA) and prepared in the desired concentration in normal sterile saline. For inducing colon cancer, mice received subcutaneous injections of DMH (25 mg/kg/week) for 12 weeks [56]. 

Mice were allocated randomly to four groups of 10 mice each: (i) Saline control, (ii) DMH-induced colon cancer control group, (iii) DMH + 100 mg/kg nitazoxanide, and (v) DMH + 200 mg/kg nitazoxanide. Daily pharmacological treatment with orally gavaged nitazoxanide was launched from the 7th week of induction of colonic cancer till the end of the therapeutic period (at the end of week 12). 

#### 4.3.4. Signaling Pathway Enrichment Analysis

Choosing the target pathway was carried out using online databases including Reactome (accessible online: http://www.reactome.org, accessed on 10 April 2021) and KEGG pathway (Accessible online: http://www.genome.jp/kegg, accessed on 10 April 2021).

#### 4.3.5. Western Blot Analysis for Wnt, β-Catenin and GSK-3β Proteins

Colonic specimens were homogenized in radioimmunoprecipitation assay (RIPA) buffer containing inhibitors for proteases and phosphatases. Then, samples were cold-centrifuged at 13,000× *g* for a period of 20-min for splitting up supernatant proteins. Then, 5 µL supernatant samples were assayed for their protein concentration by Quick Start™ Bradford Protein kit from Bio-Rad. Samples containing similar protein concentrations were boiled at 95 °C to ensure primary denaturation and then added to sodium dodecyl sulfate polyacrylamide gel. Then, the gel’s proteins were transferred to nitrocellulose membranes. Blocking of the remaining part of the membrane was done by incubating it with 5% suspension of Bio-Rad dried milk for an hour. After that, the primary antibodies were prepared in the desired concentration by dilution in Tris-buffer saline and Tween 20 (TBST). Then, the nitrocellulose membranes were subjected to incubation with the primary antibody solutions. The selected antibodies were as follows: Wnt-1 (E-10): #sc-514531 [1:1000], GSK-3β (11B9): #sc-81462 [1:1000] and actin (C-2): #sc-8432 [1:500] (SantaCruz Biotechnology, USA). In addition, β-catenin antibody (#9562) [1:1000] was obtained from Cell Signaling Technology, Inc. (USA). The next step was the incubation in the antibody solutions at 4 °C overnight with gentle vortexing. After each incubation period, the blots were washed 3 to 5 times with TBST; each wash was maintained for five minutes. Compatible horseradish peroxidase conjugated secondary antibodies were applied to the blotted proteins for 1 h. Then, the blot was washed four times using 1 × TBS, 0.1% Tween 20 Detergent (TBST) for 5 min. Visualization of the target proteins was achieved by a detection kit from Amersham BioSciences (Buckinghamshire, UK). ImageJ software (NIH) was employed to quantify the immunoreactivity based on a densitometry method and normalizing the readings against β-actin housekeeping protein.

#### 4.3.6. Inspection of Hematoxylin and Eosin-Stained Colon Specimens 

The colonic tissue sections were H&E stained, and microphotographed a digital camera (Tucsen ISH1000) on a CX23 Olympus light microscope at original magnification ×100 and ×400. Colon specimens were blindly examined for general crypt architecture and features of dysplasia (nuclear enlargement and hyperchromasia with mitoses, and loss of goblet cells. Scoring of histopathological changes was blindly carried by grading from 0–3 according to the severity of the findings. The evaluated criteria were crypt distortion, colon dysplasia, goblet cell depletion, and hyperplasia. Thereafter, the summation of the total scores was taken and presented [56]. 

#### 4.3.7. Immunohistochemistry for PCNA

4-μm sections were deparaffinized, rehydrated, and incubated with Tris-EDTA, prepared at pH = 9, for antigen retrieval. Incubation of the slides with-PCNA antibodies (NB500-106, diluted as 1:500, Novusbio) was processed overnight. The next step was by the addition of biotinylated secondary antibodies to the colon specimens for 1 h. Then, counterstaining was achieved by staining with Mayer’s hematoxylin. After that, PCNA immunostaining within the cells was imaged at ×100 and ×400. Blind quantification of the immunostaining was undertaken using the ImageJ software (NIH) for counting the PCNA positive nuclei in 4 sections imaged at ×400 from each animal. 

### 4.4. Experiment 3: In Silico Molecular Docking Simulations for Nitazoxanide

A molecular docking towards the β-catenin protein was done for demonstrating the probable interaction. First, nitazoxanide structure was optimized energetically and chemically and subjected to docking within PDB = 3SL9 whose structure was manipulated following a previous method [52]. The molecular docking calculation was validated by the molecular operating environment (MOE) 2014. Finally, the Chimera software was utilized as visualizing software to explore the drug-target interactions.

### 4.5. Statistical Analysis

Using the SPSS program, the normality of the distribution of the current data was confirmed first by the Kolmogorov–Smirnov test. Data that passed the test were analyzed employing one-way analysis of variance (ANOVA) and Tukey’s post-hoc test and presented in mean ± standard deviations. Data of colon cancer histologic grading was demonstrated in medians and quartiles and analyzed by non-parametric ANOVA. *p* < 0.05 was the accepted significance level. Inhibitory concentration 50 (IC50) values were estimated employing nonlinear regression dose-inhibition curve fit practiced by GraphPad prism 7.

## 5. Conclusions

Because the mechanism of antitumor activity of nitazoxanide was not completely studied, the present study aimed to further explore this activity focusing on the inhibitory effect on Wnt/β-catenin signaling and the consequent impact on tumor proliferation and apoptosis. 

We found that nitazoxanide robustly reduced Wnt/β-catenin-dependent tumor proliferation suggesting the promising profits of nitazoxanide in colon cancer treatment especially those with identified Wnt mutations. Future studies are warranted to confirm these results in other colon cancer cell lines and rodent models of colon cancer. In addition, clinical trials are needed to use nitazoxanide in the safe dose ranges and decide its ability to treat colon cancers.

## Figures and Tables

**Table 1:** Inhibitory concentration 50 values of nitazoxanide against cancer colon HCT-116 and normal colon (FHC) cells.

Compound	IC50 (µM)	
	HCT-116 (ATCC^®^ CCL-247™) Colon Cancer	FHC (ATCC^®^ CRL-1831™) Normal Colon
Nitazoxanide	11.07 ± 0.89	48.4 ± 1.23
5-Flurouracil	11.36 ± 0.76	>50

Data are Mean ± SD of three independent triplets. Inhibitory concentration 50 (IC50) values were estimated using nonlinear regression dose-inhibition curve fit.

**Table 2:** Summary of molecular docking stimulation of nitazoxanide.

Docked Compound	Docking Energy (Kcal/mol)	Drug-Target Interactions	Interacting Moiety
Nitazoxanide	−10.58	2 HB with Asn 290	-C = O (as hydrogen bond acceptor)
5-Flurouracil	−8.31	1 HB with Asn 290	-C = O (as hydrogen bond acceptor)
HB: hydrogen bond, mol: mole.							
A	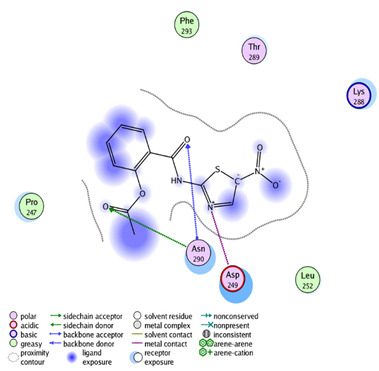	B	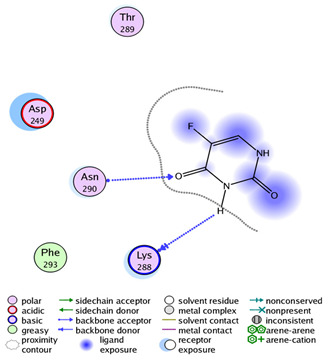
C	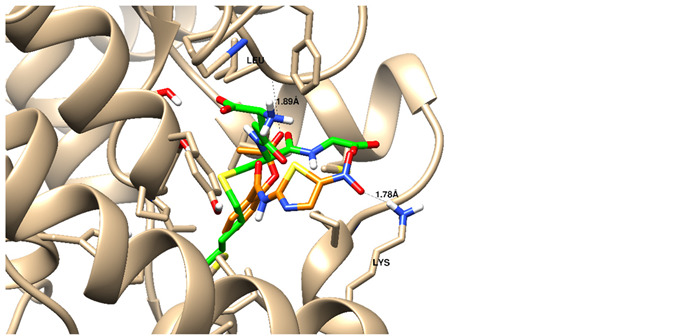		

**A** and **B:** two dimensional ligand-receptor interactions of nitazoxanide and 5-FU, respectively inside the protein pocket while **C:** Three-dimensional disposition of nitazoxanide with interactive amino acids.

**Table 3:** The set of polymerase chain reaction (PCR) primers for the selected genes.

Primer	Sequence	Annealing Temperature
**P53**	FOR: 5′-CTTTGAGGTGCGTGTTTGTG-3′ REV: 5′-GTGGTTTCTTCTTTGGCTGG-3′	57 °C
**BCL-2**	FOR: 5′-GAGGATTGTGGCCTTCTTTG-3′ REV: 5′-ACAGTTCCACAAAGGCATCC0-3′	56 °C
**BAX**	FOR: 5′-TTTGCTTCAGGGTTTCATCC-3′ REV: 5′-CAGTTGAAGTTGCCGTCAGA-3′	55 °C
**Casp-3**	FOR: 5′- TGGCCCTGAAATACGAAGTC-3′ REV: 5′- GGCAGTAGTCGACTCTGAAG -3′	56 °C
**Casp-8**	FOR: 5′- AATGTTGGAGGAAAGCAAT -3′ REV: 5′- CATAGTCGTTGATTATCTTCAGC -3′	51 °C
**Casp-9**	FOR: 5′- CGAACTAACAGGCAAGCAGC -3′ REV: 5′- ACCTCACCAAATCCTCCAGAAC -3′	58 °C
**β-actin**	FOR: 5′-GCACTCTTCCAGCCTTCCTTCC-3′ REV: 5′-GAGCCGCCGATCCACACG-3′	60 °C

## Data Availability

Data are available at MSN (in vitro study) and SAZ, NE and KMM (in vivo study) upon request.
